# Formation mechanism of overlapping grain boundaries in graphene chemical vapor deposition growth†
†Electronic supplementary information (ESI) available: Fig. S1, Table S1, calculation of formation energies of different graphene edges, calculation of the Gibbs free energy variation during the overlapping of two H terminated graphene edges, calculations on the chemical potential of H_2_ and thermodynamic diagrams. See DOI: 10.1039/c6sc04535a



**DOI:** 10.1039/c6sc04535a

**Published:** 2016-12-01

**Authors:** Jichen Dong, Huan Wang, Hailin Peng, Zhongfan Liu, Kaili Zhang, Feng Ding

**Affiliations:** a Department of Mechanical and Biomedical Engineering , City University of Hong Kong , 83 Tat Chee Avenue , Kowloon , Hong Kong , China . Email: kaizhang@cityu.edu.hk; b Institute of Textiles and Clothing , Hong Kong Polytechnic University , Kowloon , Hong Kong , China . Email: feng.ding@polyu.edu.hk; c College of Chemistry and Molecular Engineering , Peking University , Beijing 100871 , P. R. China

## Abstract

The formation of grain boundaries (GBs) in graphene films is both fundamentally interesting and practically important for many applications. A GB in graphene is known as a linear defect and is formed during the coalescence of two single crystalline graphene domains. The covalent binding between domains is broadly known as the mechanism of GB formation during graphene chemical vapor deposition (CVD) growth. Here, we demonstrate another GB formation mechanism, where two graphene domains are connected by weak van der Waals interactions between overlapping graphene layers. The formation mechanism of the overlapping GBs (OLGBs) is systematically explored theoretically and the proposed conditions for forming OLGBs are validated by experimental observations. This discovery leads to a deep understanding of the mechanism of graphene CVD growth and reveals potential means for graphene quality control in CVD synthesis.

## Abstract 2

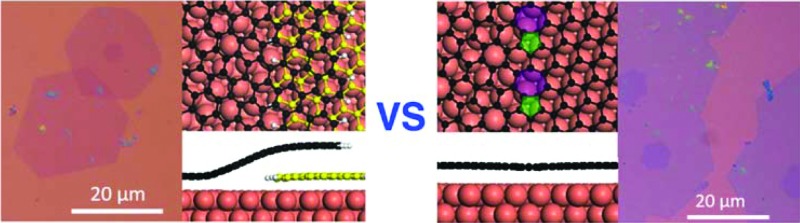
The formation mechanisms of two different types of grain boundaries (GBs), the weakly bound overlapping GB and the covalent bound GB, during graphene domain coalescence are revealed by both theoretical modeling and experimental observations.

## 1. Introduction

Various methods for the synthesis of high-quality graphene, including the mechanical exfoliation of graphite,^1^ high temperature SiC sublimation^2^ and chemical vapor deposition (CVD) growth,^3,4^ have been developed in the past 10 years in order to achieve the broad industrial applications of graphene. Among these methods, graphene CVD growth on a transition metal surface stands out because of the potential for the massive production of high quality, very large area graphene at a relatively low cost, and the existence of numerous parameters which allow for the precise tuning of the number of graphene layers and/or the achievement of controllable doping.^5–7^ In most CVD experiments, a graphene film is formed through the coalescence of a huge number of micro-sized domains. The coalescence of two graphene domains of different orientations will certainly lead to a grain boundary (GB) between them and therefore GBs in a graphene film are considered to be the main type of defects that greatly impede the quality of the graphene.^8–11^ Although the structures of GBs in graphene have been broadly studied theoretically,^12–16^ their formation mechanism during CVD growth remains unexplored.

In most CVD processes, the nucleation of graphene domains is a random process and therefore there is no control over their locations and crystalline orientations. The coalescence of two graphene domains can be achieved by forming covalent C–C bonds between them, with the disruption of the two periodic hexagonal lattices resulting in a linear defect, which is composed of pentagons and heptagons (5|7s).^13,15^ Here we term this kind of GB a covalently bonded GB (CBGB). The formation process of CBGBs has been broadly adopted to understand multicrystalline graphene film growth. However, some recent experimental observations showed evidence of another type of GB in graphene. In 2011, Robertson *et al.* found that graphene grown on Cu substrates by atmospheric pressure CVD (APCVD) with a high H_2_ partial pressure contains two types of boundaries, CBGBs and boundaries from the overlapping of graphene layers from both domains, which we term overlapping GBs (OLGBs).^17^ Afterwards, OLGBs were also observed by Tsen *et al.* ^18^ and Rao *et al.* ^19^ with both studies showing that OLGB formation has a large impact on the current across the resulting graphene sheet. These experimental evidences clearly indicate that two graphene domains can be connected either by CBGBs or OLGBs which therefore, needless to say, impose distinct differences on graphene's performance. Apart from the effect on conductance, we can anticipate that graphene with CBGBs is mechanically much stronger than graphene with OLGBs, and that the thermal conductivity through an OLGB must be much lower than that through a CBGB. Therefore, it is of great importance to unveil the formation mechanism of these two types of GBs in order to control them during graphene synthesis.

In this manuscript, the formation processes of CBGBs and OLGBs and their competition during graphene CVD growth on Cu substrates are systematically investigated. It is demonstrated that the formation of OLGBs is due to the high stability of the hydrogen passivated graphene edge and therefore the H_2_ partial pressure in the feedstock is the key experimental parameter for controlling the GB type. Considering the high formation energy of CBGBs with a misorientation angle of 10–25°, OLGBs are expected to be easily formed between graphene domains with crystalline orientation angle differences in the same range. Careful experimental studies and data in the existing literature unambiguously validate both the role of hydrogen in GB formation, and our theoretical predictions.

## 2. Computational methods

All DFT calculations in this study are performed by using the Vienna *ab initio* simulation Package (VASP).^20,21^ The exchange correlation potential is described by the Perdew–Burke–Ernzerhof implementation of the Generalized Gradient Approximation (GGA).^22^ The projector augmented wave (PAW) method is employed to treat the interaction between the valence electrons and the ion cores.^23^ Due to the unsatisfactory performance of GGA functionals in describing weak van der Waals (VDW) interactions, these calculations are corrected by the more accurate DFT-D2 method.^24^ An energy cutoff of 400 eV is adopted for the plane wave basis set.

To investigate the structural stability of the various edges and GBs in graphene grown on the Cu substrates, a slab of 3 atomic layers with the bottom layer fixed is chosen to represent the metal (111) surface. The slabs are separated by over 15 Å to eliminate interactions between the repeated images. To keep the size of the Cu slab commensurate with graphene, the lattice constant of Cu is reduced by less than 4%. The unit cell size of the substrate is 33.95 × 6.53 × 30 Å^3^, as shown in Fig. S1 in the ESI.† The formation of the graphene GB is modelled by a pair of twin graphene ribbons approaching each other, with their other sides passivated by H. All structures are optimized using the conjugate gradient method until the force on every atom is less than 0.01 eV Å^–1^. The *k*-point mesh is sampled as 1 × 5 × 1, which is tested to provide enough accuracy.

## 3. Theory

**Fig. 1:**
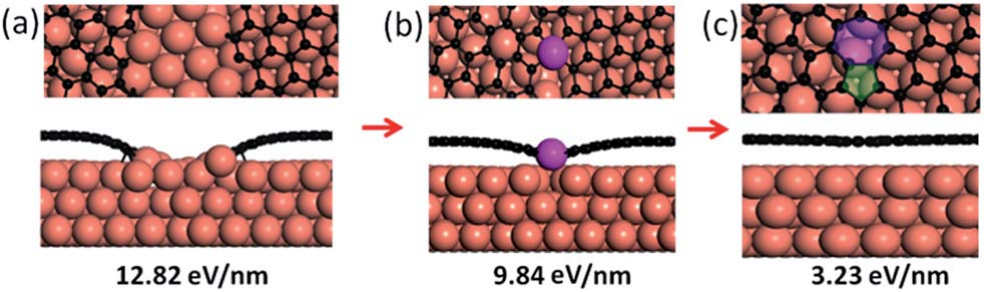
The formation process of a CBGB on a Cu(111) surface, from two distantly separated metal passivated graphene edges (a), to a partially merged GB (b) and then to a fully merged GB with pentagon–heptagon pairs (c). The formation energies for each configuration are shown as well. The C and Cu atoms are shown in black and coral, respectively. The lifted Cu atom is highlighted in magenta.

A GB in graphene has two degrees of freedom, the misorientation angle, *θ*, ranging from 0 to π/3, and the angle of alignment, *φ*, ranging from –π/6 to π/6.^13,15^ The combination of these two degrees of freedom (*θ*, *φ*) leads to a very large number of potential GB structures^15^ and therefore it is impractical to study the formation of all possible GBs. Herein, a representative GB, as shown in Fig. 1b, with *θ* = 21.8° and *φ* = 0° on a Cu(111) surface is adopted as an example to explore the formation processes of CBGBs and OLGBs.

Many experiments have shown that graphene growth is highly dependent on the partial pressure of hydrogen in the carrier gas, *P*(H_2_).^25–31^ As explained in our previous studies,^6,32^ the edge of a graphene domain can be either passivated by the transition metal surface at low *P*(H_2_) or terminated by hydrogen atoms at high *P*(H_2_).

Firstly, let us consider the coalescence of two metal passivated graphene edges. As shown in Fig. 1a–c, with a continuous attachment of C atoms, the two metal passivated graphene edges get closer and closer and spontaneously merge into a CBGB when they are in contact. It can be seen that the two distantly separated, metal passivated graphene edges (Fig. 1a) possess a very high formation energy of 12.82 eV nm^–1^. Then, when the two edges are sufficiently close to each other, C–C bonds form between them spontaneously (Fig. 1b) and some metal atoms (highlighted in magenta) are lifted from the substrate to passivate the unsaturated σ bonds near the graphene boundary. The partially merged boundary has a much lower formation energy than the distantly separated metal passivated graphene edges, 9.84 eV nm^–1^, which indicates that the formation of C–C bonds at the boundary is energetically preferred. When more C atoms are added to the GB, a complete CBGB in a sp^2^ carbon network is formed and all of the lifted metal atoms automatically migrate back to their original positions in the substrate (Fig. 1c). The final CBGB has a very low formation energy of only 3.23 eV nm^–1^. The substantial reduction in the formation energy strongly suggests that a CBGB can be spontaneously formed when two metal passivated graphene edges meet each other. It is important to note that, depending on the atomic structure, the formation energy of a CBGB may vary in the range from 1.0 to 9.0 eV nm^–1^,^12–16^ which means that the maximum formation energy of a CBGB is smaller than the formation energy of two metal passivated graphene edges on a Cu surface. So, we can conclude that CBGBs can be easily formed during the coalescence of two grains with metal passivated graphene edges. Previous theoretical explorations have undoubtedly demonstrated that metal passivated graphene edges are energetically more preferred in graphene CVD growth on active catalyst surfaces (such as Ni, Co, Pt, Pd, Ru, Rh, Ir, *etc.*) at high temperature and with low *P*(H_2_),^32,33^ so we can anticipate that the experimental condition for forming CBGBs is the same.

**Fig. 2:**
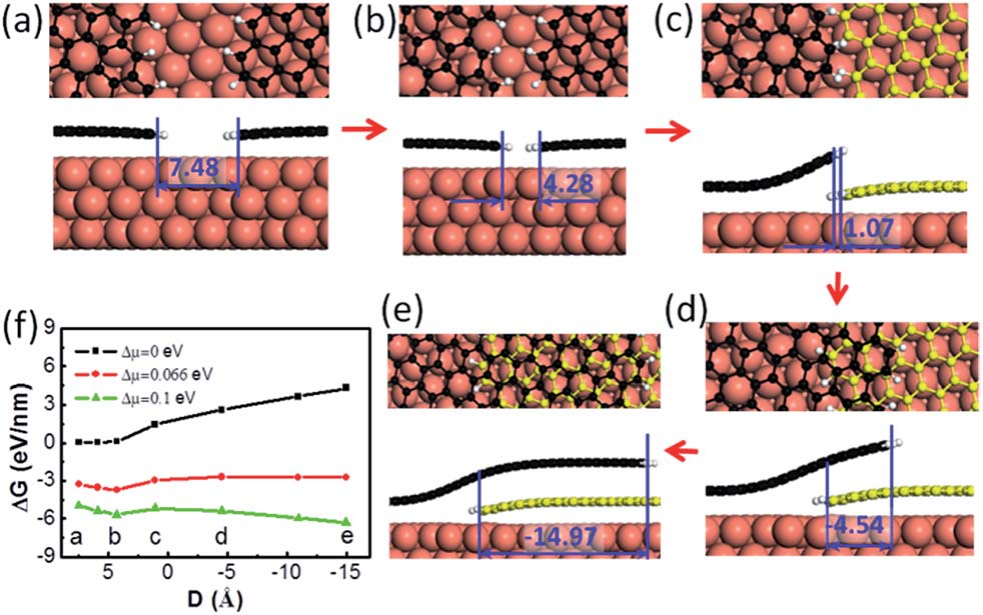
(a–e) The formation process of overlapping grain boundaries (OLGBs) from two H terminated graphene edges on the Cu(111) surface. The H atoms are shown in white. The edge distance is given in Angstroms. (f) The Gibbs free energy of (a–e) at different driving forces for the graphene growth (the chemical potential differences between carbon in the feedstock and graphene) as a function of the distance, *D*, between the two edges, where the negative values represent the overlapping configuration.

Next, let us consider the coalescence of two graphene domains with H terminated edges, which is known to be crucial for understanding graphene CVD growth.^32^ The growth of two H terminated graphene edges on a Cu(111) surface is shown in Fig. 2a–e. It can be clearly seen that the meeting of two H terminated graphene edges (Fig. 2b & c) does not lead to covalent binding between them because all of the dangling bonds at the edges are saturated by H atoms. Under such conditions, forming a covalent bond between the passivated graphene edges means that the system must overcome a high activation energy and therefore two such H terminated graphene edges may pass over each other easily to form an OLGB (Fig. 2c–e). Fig. 2f presents the Gibbs free energy variation during the overlapping of the two H terminated graphene edges under different driving forces (Δ*μ*), which is defined as the chemical potential difference between a carbon atom in the feedstock and in graphene (see ESI†). One can see that the barrier that prevents the two H terminated graphene edges from passing over each other is very low (*e.g.*, only 0.52 eV nm^–1^ for a small driving force of Δ*μ* = 0.1 eV per C atom). This is expected because the formation of an overlapping GB only involves the variation of the very weak van der Waals interactions and a slight bending of the graphene. So we can conclude that the formation of the OLGB is very possible during graphene CVD growth if the edges of the graphene domains are terminated by H atoms.

The above analysis shows us that the formation of H terminated graphene edges is a precondition for forming OLGBs, and thus the stability of H terminated graphene edges *versus* that of metal passivated graphene edges is crucial for the formation of OLGBs. As demonstrated in previous studies, the Gibbs free energy difference between an H terminated graphene edge and the corresponding metal passivated graphene edge is^6,32^

1 Δ*G* = Δ*E*_F_ + Δ*F*_V_ – *N*_H_ × *μ*_H_(*T*,*P*)

where Δ*E*_F_ = *E*_F_(HTGE) – *E*_F_(MPGE) is the formation energy difference between an H terminated graphene edge and a metal passivated graphene edge, Δ*F*_V_ is the vibrational free energy of the H atoms at the H passivated graphene edge, *μ*_H_ is the chemical potential of H_2_ in the gas phase as a function of temperature, *T*, and *P*(H_2_), and *N*_H_ is the number of H atoms. The details of the calculation are provided in the ESI.†

**Fig. 3:**
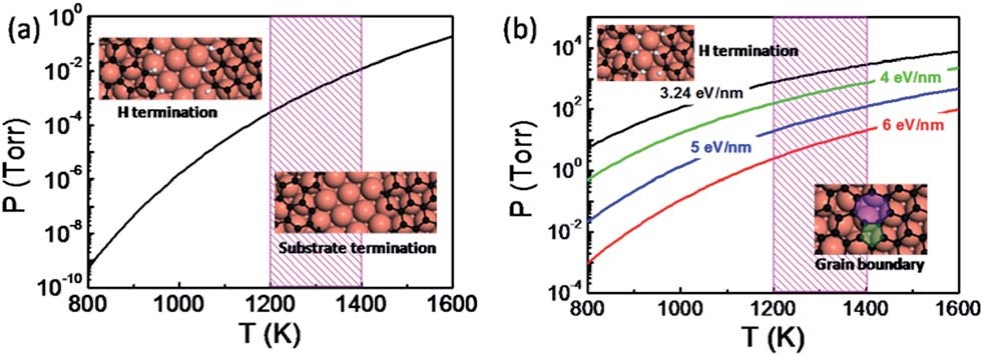
(a) Thermodynamic diagram between an H terminated graphene edge and a metal passivated graphene edge on a Cu(111) substrate. (b) Thermodynamic diagram between the overlapping grain boundary (OLGB) and the covalently bonded grain boundary (CBGB) on the Cu(111) surface, where different formation energies of the CBGB are adopted and the OLGB is represented by the modelled structure. The typical temperature range for graphene CVD growth is shaded.

The thermodynamic diagram of the specific H terminated graphene edge and the metal passivated graphene edge on the Cu(111) surface can be obtained by solving eqn (1) and is plotted in Fig. 3a. At a typical temperature for graphene CVD growth (*T* = 1300 K) the metal passivated graphene edge is more stable than the H terminated graphene edge even if the hydrogen partial pressure *P*(H_2_) < 10^–3^ Torr and therefore the coalescence of two metal passivated graphene edges would lead to a CBGB. In contrast, the H terminated graphene edge prevails at lower temperatures or higher *P*(H_2_) values and the coalescence of the two hydrogen terminated graphene domains may lead to an OLGB.

It is important to note that two H terminated graphene edges might merge into a CBGB because graphene growth always involves the fracture and reforming of C–C and C–H bonds at the edge. In the case that a CBGB is energetically more favorable than a corresponding OLGB, two H terminated graphene edges certainly have the probability of being welded into a CBGB. To include this probability, let us compare the Gibbs free energy of a CBGB and a corresponding OLGB (see ESI†). Fig. 3b shows the thermodynamic diagram of CBGBs and OLGBs, where the OLGB is represented by two H terminated graphene edges which are nearly in contact with each other. It is known that the formation energy of CBGBs may vary in a large range and therefore we presented the diagram with CBGB formation energies of 3.24, 4.0, 5.0 and 6.0 eV nm^–1^. As expected, it can be clearly seen that the OLGB is increasingly preferred if the formation energy of the CBGB is higher.

## 4. Experiments

We carried out experiments to verify the above predictions. We grew graphene under both H_2_ rich and H_2_ poor conditions. Graphene samples were grown on copper foil (25 μm, 99.8%, Alfa Aesar) using low-pressure CVD (LPCVD). Before growth, the copper foils were electropolished according to the method reported by Han *et al.* ^34^ Then, the copper foil was loaded into the hot center of the furnace and heated to 1000 °C in a 350 sccm H_2_ atmosphere with a pressure of 500 Pa within 30 min and annealed under this temperature for 2–4 h to enlarge the domain sizes of the polycrystalline copper foil. The H_2_ poor CVD experiments were carried at a H_2_ partial pressure of ∼3.7 × 10^–2^ Torr and the specific experimental parameters are 5 sccm H_2_, 1 sccm CH_4_, and 300 sccm Ar with the system pressure kept at 500 Pa for 20 min. The H_2_ rich experiments were carried with a H_2_ partial pressure of ∼10 Torr and the experimental parameters were 1 sccm CH_4_, and 800–900 sccm H_2_, and the system pressure was kept at 1200–1500 Pa for 1 h. The graphene samples were transferred onto a 300 nm SiO_2_/Si substrate using a wet-etching method with the help of polymethyl methacrylate (PMMA) for characterization.

**Fig. 4:**
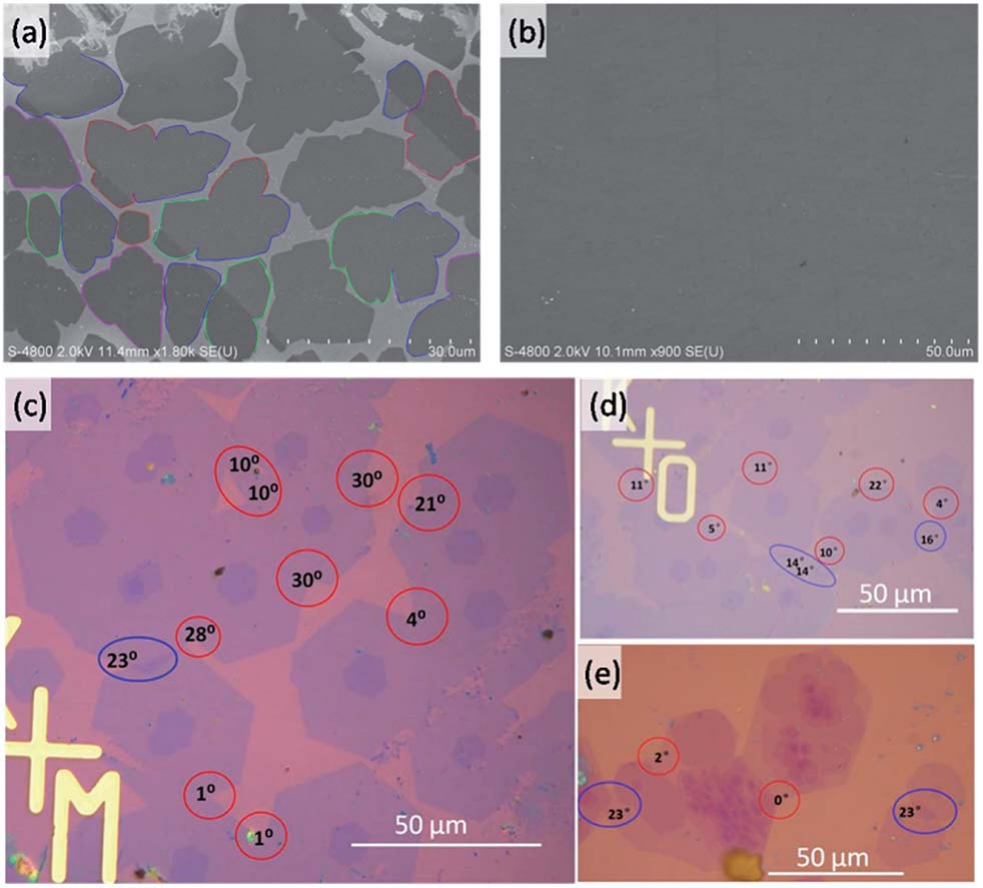
(a and b) Scanning electron microscope (SEM) images of graphene monolayers grown on Cu substrates under low H_2_ partial pressures with a growth time of 5 and 10 min, respectively. The boundaries of graphene domains contacting each other are denoted by colored lines. (c–e) Optical images of the graphene domains grown under high H_2_ partial pressures. The GB misorientation angles are given. CBGBs and OLGBs are denoted by red and blue circles, respectively.

For graphene grown under low H_2_ partial pressures (Fig. 4a and b), many domain coalescences were observed after a growth time of 5 min (Fig. 4a). It is clear that there is no contrast variation in area between the two merged domains. Therefore, we concluded that all of the observed GBs between the domains are CBGBs. A complete graphene film is formed after 10 min of growth (Fig. 4b). The whole graphene film shows very even contrast except for a few wrinkles, which further evidences that no OLGBs are formed during graphene growth at low H_2_ partial pressures. This result validates our theoretical prediction that, under low H_2_ partial pressures, graphene edges are terminated by the metal substrate and the active edge atoms will form CBGBs during the coalescence of two graphene domains.

In contrast, both CBGBs and OLGBs were observed in graphene grown under a high H_2_ partial pressure (Fig. 4c–e). The coexistence of CBGBs and OLGBs is also consistent with our theoretical predictions outlined above. The orientation of a graphene domain can be characterized by its straight edges, which are believed to lie along the zigzag crystalline direction of the graphene's hexagonal lattice. Interestingly, it is found that the type of GB depends on the misorientation angle between two domains and the OLGBs having a higher population in the angle range between 15 and 20 degrees, which will be explained below.

**Fig. 5:**
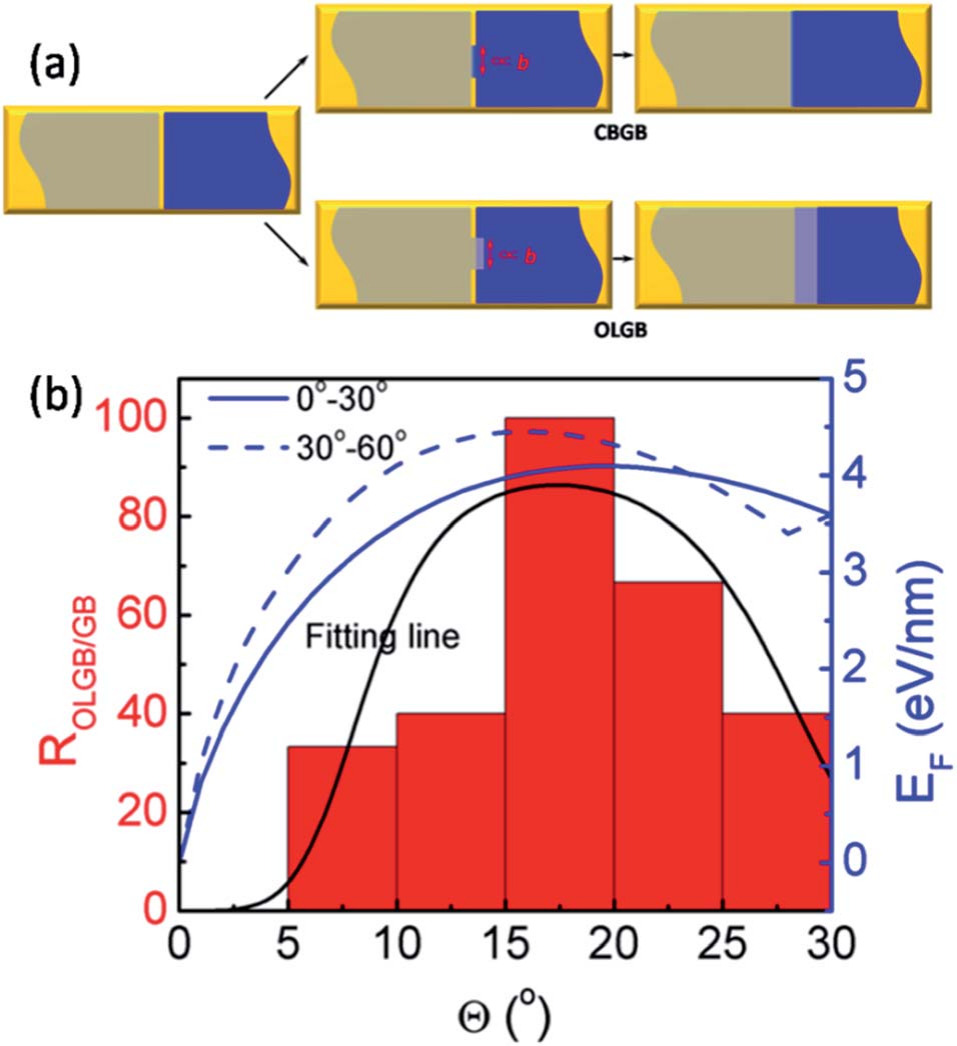
(a) Schematics of the formation of CBGBs and OLGBs between two neighboring graphene domains (denoted by the gray and blue colors) on a substrate (denoted by the gold color). The GB nucleation size is proportional to graphene lattice constant *b*. (b) The ratio of OVGBs to all GBs (OLGBs and CBGBs) in CVD grown graphene on Cu foil in an H_2_ rich environment and the fitted formation energies of CBGBs as a function of the misorientation angle, where the two fitting lines correspond to the true misorientation angle range of (0°, 30°) and (30°, 60°), respectively (ref. 15). The black line is fitted from eqn (3).

In order to more deeply understand the dependence of the GB type on the misorientation angle, let us explore the formation process of CBGBs and OLGBs during graphene CVD growth under high H_2_ pressures. During growth, two neighbouring graphene domains approach each other but they will not merge into a CBGB abruptly because both domain edges are H terminated and inert. Under such a circumstance, whether the formed GB could be either a CBGB or an OLGB is determined by the next step growth of one of the edges. If next step growth of an edge forms a nucleus which stitches the two grains together by a covalent bond, then a CBGB will be formed naturally. While, in the case where the extruder of an edge moves to the top of the other edge, a OLGB will eventually be formed (Fig. 5a). The nucleation steps for both CBGBs and OLGBs are random events; which one will occur depends on their barrier, which can be approximated to be proportional to *E*_CBGB_ × *b* and *E*_OLGB_ × *b*, respectively, where *E*_CBGB_ and *E*_OLGB_ are the formation energies of the CBGB and the OLGB, and *b* = 0.246 nm represents the size of the nucleus, which is estimated as the lattice constant of graphene. It is broadly reported that the formation energies of hydrogen passivated graphene edges are nearly independent of the edge type,^32^ while the formation energies of CBGBs are highly misorientation angle dependent. Therefore, the ratio of the probabilities of forming an OLGB and a CBGB is given by:2
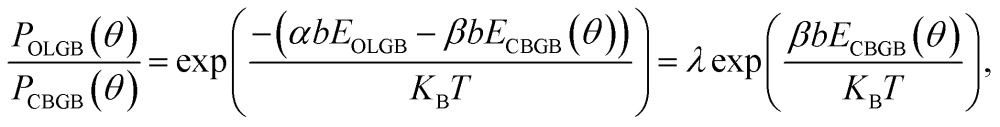
where *αbE*_OLGB_ and *βbE*_CBGB_ are the nucleation barriers for OLGBs and CBGBs, respectively, *α* and *β* are constants, 
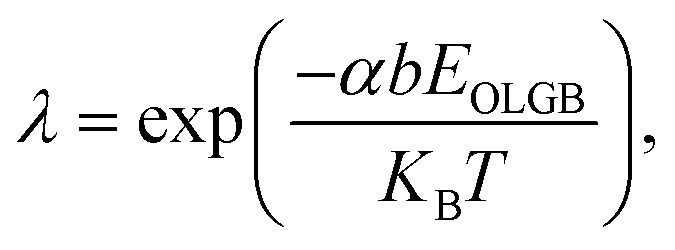
 *K*_B_ is the Boltzmann constant, and *T* is the temperature. Considering the uniformity of the probabilities, the probability of forming a CBGB is:3
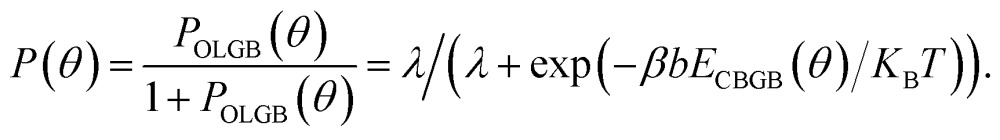



Recent studies have shown that the misorientation angle dependent formation energy of CBGBs shows an “M” shape, with two peaks appearing in the ranges of (10°, 25°) and (35°, 50°) respectively, ranging from 0° to 60° (Fig. 5b. Details are shown in Fig. 5 of ref. 15).^12–15^ Therefore, one can anticipate that OLGBs can be easily formed if the misorientation angle between the two graphene domains is in the range of (10°, 25°) and (35°, 50°). From an experimental perspective, the measured misorientation angle of a GB is usually folded in the range of (0°, 30°) because it is impractical to identify the true misorientation angles *θ* from 60°–*θ* without atomic resolution (Fig. 4c–e). Therefore, experimentally, we can only expect one population peak at *θ* ∈ (10°, 25°) in the observed misorientation angle range of *θ* ∈ (0°, 30°). To verify this prediction, we counted the numbers of OLGBs and CBGBs in Fig. 4c–e. With some data extracted from the literature (see Table S1 in the ESI†), the ratio of OLGBs to all GBs *versus* the misorientation angle is shown in Fig. 5b, from which a peak can be undoubtedly seen in the range of 10° to 25°, exactly as predicted by our analysis. We can further quantitatively predict the proportion of OLGBs among all GBs by fitting the above experimental statistics to eqn (3). Assuming the graphene growth temperature *T* is 1300 K, *β* and *λ* are then found to be 1.24 × 10^–5^ and 12.6, respectively. Clearly, the fitting line shown in Fig. 5b is strongly consistent with the experimental data.

It is worth mentioning that the coalescence of two domains with metal passivated graphene edges must lead to a CBGB due to the high activity of the edge C atoms and the high instability of the metal passivated graphene edges. Previous studies have demonstrated that most catalysts, such as Ni, Co, Pt, Pd, Rh, Ir, and Ru, are more active than Cu and the metal passivated graphene edges on these surfaces have much lower formation energies than that on Cu.^32,33^ So, OLGBs were rarely observed for graphene growth on these catalysts because of the high stability of metal passivated graphene edges.

## 5. Conclusions

In summary, we performed a systematic theoretical study on the formation mechanism of overlapping grain boundaries (OLGBs) during graphene CVD growth, including the thermodynamic stability and the formation processes. This study reveals that the hydrogen passivation of the graphene edge is a precondition of forming OLGBs, and therefore OLGBs might be formed during graphene CVD growth on a less active catalyst surface, with high hydrogen partial pressure and high temperature. Further analysis reveals that an OLGB can be easily formed during the coalescence of two graphene domains with crystalline orientation differences in the range of 10 to 25 degrees, due to the high instability of the CBGBs. Furthermore, both our experimental study and literature data validated the proposed experimental conditions for OLGB growth and the theoretical prediction of the abundance of OLGBs in the misorientation angle range of 10 to 25 degrees. This study explains well the mystery of forming the OLGBs observed in previous graphene synthesis experiments, and leads to a deeper insight into graphene CVD growth. It also allows us to achieve the desired graphene growth by rational experimental design.
