# Supplementary material for: Formation mechanism of overlapping grain boundaries in graphene chemical vapor deposition growth
Source: Chem Sci. 2016 Dec 1;8(3):2209–14. doi: 10.1039/c6sc04535a (PMC5408562; doi:10.1039/c6sc04535a)
Supplement: Supplementary file 1 [file SC-008-C6SC04535A-s001.pdf]

Electronic Supplementary Material (ESI) for Chemical Science.
This journal is © The Royal Society of Chemistry 2016

Electronic Supplementary Information

Formation Mechanism of Overlapping Grain Boundaries in Graphene Chemical Vapor Deposition Growth

Jichen Dong,[a,b] Huan Wang,[c] Hailin Peng,[c] Zhongfan Liu,[c] Kaili Zhang,[a,]* Feng Ding[b,]*

[a] Department of Mechanical and Biomedical Engineering, City University of Hong Kong, 83 Tat Chee Avenue, Kowloon, Hong Kong

[b] Institute of Textiles and Clothing, Hong Kong Polytechnic University, Kowloon, Hong Kong

[c] College of Chemistry and Molecular Engineering, Peking University, Beijing 100871, P. R. China

*Corresponding Authors: kaizhang@cityu.edu.hk; feng.ding@polyu.edu.hk

1. Substrate model

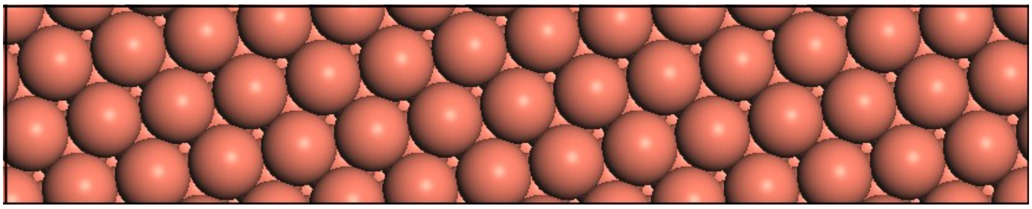

Fig. S1 Unit cell of the Cu(111) substrate.

2. Calculations on the formation energy of different edges

As a reference, the formation energy of the HTGEs (When the two neighboring graphene ribbons are far apart from each other with their edges terminated by H) is first obtained:

$$E_{F-H} = (E_T - E_{Sub} - N_C \times \varepsilon_C - N_H \times \varepsilon_{H_2} / 2) / 4l\,, \qquad (1)$$

where E_T is the total energy of the structure, E_{Sub} is the energy of the metal substrate, ε_C and ε_{H2} represent the energy of graphene on substrate per atom and the energy of a H_2 molecule, N_C and N_H denote the number of C and H atoms in the structure, and l is the grain boundary length. 4l is used to account for the four H terminated edges of the of two graphene ribbons in the model.

For the formation energies of CBGB and MPGE, they are calculated as:

$$E_F = (E_T - E_{Sub} - N_C \times \varepsilon_C - N_H \times \varepsilon_{H_2} / 2) / l - 2 \times E_{F-H}\,, \qquad (2)$$

where the second term at the right side is used to cancel out the effect of the H passivated edges of other sides of graphene ribbonds.

3. Calculation on the Gibbs free energy variation during the overlapping of two HTGEs

The Gibbs free energy change during the overlapping of two HTGEs is defined as

$$\Delta G(N_C)=E_F(N_C)-N_C\times\Delta\mu \quad (3)$$

where $E_F(N_C)$ is the formation energy of the system, N_C is the number of C atoms and $\Delta\mu$ is the chemical potential difference between C source and graphene on substrate. The formation energy of the system is defined by equation (2).

.

4. Calculations on the chemical potential of H_2 and thermodynamic diagrams

The H chemical potential μ_H as a function of temperature T and pressure P is obtained by[1]:

$$\mu_H = [E_{H_2} - k_B T \ln(g k_B T / P \times \xi_{trans}\xi_{rot}\xi_{vibr})]/2, \quad (4)$$

where E_{H2} is the energy of a H_2 molecule, k_B is the Boltzmann constant, g is 2 (accounting for the degree of degeneracy of the electron energy level), ξ_{trans}, ξ_{rot} and ξ_{vibr} are the partition functions of translation, rotation and vibration motions.

For calculating the thermodynamic diagrams between H passivated grain boundary and substrate passivated grain boundary, the vibration entropy ΔF_V is obtained by

$$\Delta F_V = -k_B T[\frac{\beta\hbar\omega}{e^{\beta\hbar\omega}-1} - \ln(1-e^{-\beta\hbar\omega})], \quad (5)$$

where β equals $1/k_BT$, ω is the vibration frequency of the H atoms at the edges.[2]

The thermodynamic diagrams between H passivated grain boundary and covalent bonding mediated grain boundary are obtained by calculating the Gibbs free energy difference ΔG as:

$$\Delta G = \Delta E_T + \Delta F_V - N_H \times \mu_H(T,P), \quad (6)$$

where ΔE_T is the total energy difference between the two structures, and the vibration entropy ΔF_V is taken by the vibration entropy difference between the H passivated edges and the covalent bonding mediated grain boundary.

Table S1 Statistics on the angle and type of graphene GBs in our experiments and other references.

Number	Angle	OLGB or not
1	1°	no
2	1°	no
3	1°	no
4	2°	no
5	4°	no
6	4°	no
7	5°	no
8[a]	8°	yes
9	10°	no
10	10°	no
11	10°	no
12	11°	no
13	11°	no
14	14°	yes
15	14°	yes
16	16°	yes
17	21°	no
18	22°	no
19	23°	yes
20	23°	yes
21	23°	yes
22[a]	24°	yes
23[a]	26°	yes
24[a]	28°	Yes
25	28°	no
26	30°	no
27	30°	no

[a] These data are taken from Figures 3, 6 and 8 and in Ref. 3 and Figure S7 in Ref. 4.

References

1 L. D. Landau, E. M. Lifshitz, Statistical Physics, Butterworth, Oxford, 1995.

2 K. Reuter, M. Scheffler, *Phys. Rev. B* 2001, **65**, 035406.

3 A. W. Robertson, A. Bachmatiuk, Y. A. Wu, F. Schäffel, B. Rellinghaus, B.

Büchner, M. H. Rümmeli, J. H. Warner, *ACS nano* 2011, **5**, 6610.
4 A. W. Tsen, L. Brown, M. P. Levendorf, F. Ghahari, P. Y. Huang, R. W. Havener, C. S. Ruiz-Vargas, D. A. Muller, P. Kim, J. Park, *Science* 2012, **336**, 1143.
